# The Caspase Inhibitor Z-VAD-FMK Alleviates Endotoxic Shock via Inducing Macrophages Necroptosis and Promoting MDSCs-Mediated Inhibition of Macrophages Activation

**DOI:** 10.3389/fimmu.2019.01824

**Published:** 2019-08-02

**Authors:** Xuehui Li, Xiaoying Yao, Yuzhen Zhu, Hui Zhang, Haiyan Wang, Qun Ma, Fenglian Yan, Yonghong Yang, Junfeng Zhang, Hui Shi, Zhaochen Ning, Jun Dai, Zhihua Li, Chunxia Li, Fei Su, Yin Xue, Xiangzhi Meng, Guanjun Dong, Huabao Xiong

**Affiliations:** ^1^Cheeloo College of Medicine, Shandong University, Jinan, China; ^2^Institute of Immunology and Molecular Medicine, Jining Medical University, Jining, China; ^3^Department of Central Laboratory, Affiliated Hospital of Jining Medical University, Jining, China; ^4^Institute of Animal Husbandry and Veterinary Sciences, Zhejiang Academy of Agricultural Sciences, Hangzhou, China; ^5^Zhejiang Provincial Center for Disease Control and Prevention, Hangzhou, China; ^6^Department of Microbiology, Immunology, and Molecular Genetics, University of Texas Health Science Center at San Antonio, San Antonio, TX, United States; ^7^Department of Medicine, Icahn School of Medicine at Mount Sinai, Precision Immunology Institute, New York, NY, United States

**Keywords:** zVAD, necroptosis, macrophage, MDSCs, endotoxin shock

## Abstract

Macrophages play a critical role in the pathogenesis of endotoxin shock by producing excessive amounts of pro-inflammatory cytokines. A pan-caspase inhibitor, zVAD, can be used to induce necroptosis under certain stimuli. The role of zVAD in both regulating the survival and activation of macrophages, and the pathogenesis of endotoxin shock remains not entirely clear. Here, we found that treatment of mice with zVAD could significantly reduce mortality and alleviate disease after lipopolysaccharide (LPS) challenge. Notably, in LPS-challenged mice, treatment with zVAD could also reduce the percentage of peritoneal macrophages by promoting necroptosis and inhibiting pro-inflammatory responses in macrophages. *In vitro* studies showed that pretreatment with zVAD promoted LPS-induced nitric oxide-mediated necroptosis of bone marrow-derived macrophages (BMDMs), leading to reduced pro-inflammatory cytokine secretion. Interestingly, zVAD treatment promoted the accumulation of myeloid-derived suppressor cells (MDSCs) in a mouse model of endotoxin shock, and this process inhibited LPS-induced pro-inflammatory responses in macrophages. Based on these findings, we conclude that treatment with zVAD alleviates LPS-induced endotoxic shock by inducing macrophage necroptosis and promoting MDSC-mediated inhibition of macrophage activation. Thus, this study provides insights into the effects of zVAD treatment in inflammatory diseases, especially endotoxic shock.

## Introduction

Endotoxic shock is a pathological condition, in which a patient experiences systemic inflammation, organ dysfunction, and organ failure. It is initiated by the dysregulation of cytokine production, also known as cytokine storm (1). Both of endogenous macrophages and macrophages in the abdominal cavity have been established to play a pivotal role in the pathogenesis of endotoxin shock (2–5). In the early stages of endotoxin shock, hyper-activated macrophages can drive tissue damage by producing excessive amounts of pro-inflammatory cytokines. After stimulation with bacterial lipopolysaccharide (LPS), macrophages can differentiate into classically macrophages (M1) that can secrete pro-inflammatory cytokines, such as tumor necrosis factor-α (TNF-α), interleukin (IL)-6, and IL-12, and produce nitric oxide (NO) (6–11). In the early stages of endotoxin shock, LPS can bind TLR4 and trigger MyD88- and TRIF-dependent expression of pro-inflammatory cytokines and type I interferons (12–14). Therefore, to understand the pathogenesis and improve the treatment of endotoxic shock, it is important to investigate the mechanisms that regulate the survival, accumulation, and activation of macrophages.

Necroptosis is a programmed form of necrosis, also known as inflammatory cell death. It is regulated by a specific signaling pathway that depends on receptor interaction protein kinases 1 and 3 (RIP1 and RIP3) and mixed lineage kinase domain-like (MLKL) (15–18). The compound z-VAD-FMK (zVAD), a pan-caspase inhibitor that prevents apoptosis in many different cell types, can trigger necroptosis by inhibiting the activity of caspase-8, which can be triggered by many inflammatory stimuli including Toll-like receptor 3 and 4 agonists, TNF-α, and certain viral infections (19). Necroptosis has recently emerged as an important pathological driver of inflammatory diseases (20). To date, the effect of zVAD-mediated necroptosis in the regulation of inflammatory diseases has remained controversial and zVAD may play multiple roles in inflammatory diseases (21). Although many studies have shown that zVAD-mediated necroptosis contributes to the pathogenesis of inflammation, some other studies indicated that necroptosis plays an anti-inflammatory role by preventing specific viral and bacterial infections, and could contribute to tissue generation (22, 23). For example, zVAD showed positive effect on lung injury in the severe acute pancreatitis model (SAP) in rats, but had a negative effect in severe pneumovirus disease in mice (24, 25). In addition, zVAD aggravated renal function and facilitated autophagy in cisplatin acute kidney injury (AKI) (26). However, it has remained unknown whether zVAD can modulate the activity of macrophages in the pathogenesis of endotoxin shock and its associated pathological conditions.

The inhibition of LPS-induced pro-inflammatory responses in macrophages can clearly help to ameliorate endotoxic shock. Based on recent studies, certain immunoregulatory cells, cytokines, and small molecules that can regulate LPS-induced pro-inflammatory responses in macrophages have shown the capacity to alleviate endotoxin shock (27–30). Studies by ourselves and others found that MDSCs, a novel heterogeneous population of immature myeloid cells that plays a critical role in both innate and adaptive immunity, accumulate during the onset of endotoxin shock. MDSCs help to control the inappropriate activation of inflammation and alleviate disease by inhibiting the polarization of M1 macrophages (31, 32). Additionally, MDSCs can act as an immune suppressor by producing high levels of immunosuppressive mediators, including Arginase-1 (Arg-1), inducible nitric oxide synthase (iNOS), and IL-10 (33). In mice, MDSCs can be broadly characterized as CD11b^+^Gr-1^+^ cells. Specifically, MDSCs can be divided into two subtypes: granulocytic MDSCs (G-MDSCs) and monocytic MDSCs (M-MDSCs), which are identified with a CD11b^+^Ly6G^+^Ly6C^low^ or CD11b^+^Ly6G^−^Ly6C^high^ phenotype, respectively (34). Thus, regulation of the proliferation and function of MDSCs can significantly affect the activation of immune responses and the pathogenesis of inflammatory diseases. However, it remains unclear whether zVAD can regulate LPS-induced pro-inflammatory responses in macrophages directly or through the accumulation of MDSCs, and thereby affect the pathogenesis of endotoxic shock.

In this present study, we investigated the effects of zVAD on the pathogenesis of endotoxic shock as well as macrophages activation and necroptosis. We found that intraperitoneal injection of zVAD markedly reduced the mortality rate of mice against LPS challenge and alleviated LPS-induced liver and lung pathology. Moreover, intraperitoneal injection of zVAD significantly reduced the concentration of inflammatory cytokines in serum by promoting necroptosis of peritoneal macrophages and suppressing macrophage activation *in vivo*. Our *in vitro* studies showed that zVAD blocked the LPS-induced secretion of inflammatory cytokines in BMDMs by causing the necroptosis of macrophages, a process in which NO played an important regulatory role. Moreover, we found that the intraperitoneal injection of zVAD significantly promoted the aggregation of MDSCs in mice undergoing endotoxin shock, which may have contributed to the inhibition of M1 macrophage activation. Taken together, our studies reveal a critical role of zVAD in alleviating the pathogenesis of endotoxic shock, which could provide a novel basis for the treatment of endotoxic shock.

## Materials and Methods

### Animals

Female C57BL/6 mice, 6–8 weeks old, were obtained from Peng Yue experimental animal breeding company (Jinan, China) and were housed in the animal facilities under specific pathogen-free conditions at Jining Medical University. iNOS^−/−^ mice were obtained as a gift from professor Tang Hua in Taishan Medical College. All the mice were maintained under specific pathogen-free conditions at Jining Medical University and used at 6–8 weeks old. All animal experiments were carried out in accordance with protocols approved by the Institutional Animal Care and Use Committees of the Animal Care Committee at Jining Medical University.

### Cell Viability Assay

BMDMs and peritoneal macrophages at the density of 1 × 10^5^ were seeded into a 96-well plate and incubated with different concentrations of zVAD for 48 h. The cells were treated with 10 μl Cell Counting Kit-8 (CCK8) reagents for an additional 1 h at 37°C in the dark. The absorbance at 450 nm was measured in a microplate reader (BioTek).

### Preparation of Bone Marrow-Derived Macrophages

The bone marrow cells were rinsed out from tibias and femurs of the mouse with phosphate buffered saline (PBS), and then the cells were planted in complete DMEM supplemented with GM-CSF (10 ng/ml; PeproTech, USA). After 3 days, all the medium was refreshed by DMEM supplemented with GM-CSF (10 ng/ml). After 7 days, the bone marrow-derived macrophages (BMDMs) can be obtained and used for subsequent experiments. In this study, the cells were pretreated with zVAD (0, 20, 40, and 80 μM, Beyotime Biotechnology, China) for 30 min followed by stimulation of LPS (100 ng/ml).

### Isolation of G-MDSCs Purification and G-MDSCs Suppressive Assay

Spleen-derived G-MDSCs were purified from LPS or LPS plus zVAD treated C57BL/6 mice using a Myeloid-Derived Suppressor Cell Isolation Kit (Miltenyi Biotec). BMDMs (2 × 10^5^ cells/well) were co-cultured with purified G-MDSCs for 12 h and then stimulated with 100 ng/ml LPS. The cells were cultured for 24 h before being analyzed by flow cytometry.

### Preparation of Peritoneal Macrophages

2 ml 3% thioglycollate medium (sigma, USA) was injected into the abdominal cavity of mice. After 3 days, the peritoneal cells were rinsed out with PBS and were planted on the medium subsequently. The suspended cells were discarded after 3 h and the medium was replaced by fresh DMEM. In this study, the adherent peritoneal macrophages were pretreated with zVAD (0, 5, 15, and 45 μM) for 30 min followed by challenge of LPS (100 ng/ml).

### Murine Model of Endotoxic Shock

Female WT and iNOS^−/−^ mice, 6–8 weeks old, were randomly allotted to different groups, respectively. The mice were pretreated or post-treated with zVAD (5, 10, and 20 μg/g of body weight) or vehicle (saline) for 2 h and endotoxic shock was induced by an intraperitoneal injection of LPS (10 μg/g of body weight) and saline was used as control. Serum samples were collected after 6 h and livers, lungs, and spleens were collected after 12 h, peritoneal cells were collected for PI detection after 6 and 12 h. As for the experiment to record the fatality rate of mice, the mice received an intraperitoneal injection of LPS (25, 37.5, or 50 μg/g of body weight) and the survival of mice were monitored every hour.

### Quantitative Real-Time PCR Analysis

Total RNA was extracted using Trizol (Invitrogen, Carlsbad, USA) according to the manufacturer's instructions. Quantitative real-time PCR (qPCR) assays of mRNA were carried out using SYBR Green PCR Master Mix. The reactions were incubated in a 96-well plate at 95°C for 5 min followed by 40 cycles of 95°C for 15 s, 60°C for 30 s, and 72°C for 30 s. The 2^−ΔΔ*CT*^ formula was used to calculated the relative gene expression, with GAPDH as an internal control. All experiments were practiced in triplicate.

### Immunofluorescence Staining

After dewaxing, hydration and antigen retrieval, the paraffin sections were washed with PBS and then 3% H_2_O_2_ were used for 20 min to block endogenous enzymes. After washing, slides were blocked with 1% bovine serum albumin (BSA) for 30 min. Then, sliders were incubated with primary antibodies overnight at 4°C. The next day, slides were exposed to fluorochrome-labeled secondary antibodies for 1 h (25°C) after washing with PBS. In the end, the cover slips were sealed with an anti-fluorescence quenching agent.

### MPO Activity Assay

MPO activity was measured in liver and lung samples through use of the MPO Activity Detection Kit (Nanjing Jiancheng). To be short, ~50 mg tissue samples were homogenized in homogenization buffer (provided in kit) to determine MPO activity from 36 separate animals as described in Murine Model of Endotoxic Shock.

### H&E Staining

Sections (4 μm) were cut from paraffin-embedded lung and liver tissues, fixed in 4% paraformaldehyde (Sigma, USA) for 24 h. The slides were stained with hematoxylin and eosin and were observed under an optical microscope. The degree of lung injury was evaluated based on the following histological features: hemorrhage, lung edema, inflammatory cell infiltration, hyaline membrane, and atelectasis. The degree of each item was graded numerically from 0 (normal) to 4 (diffuse injury) according to the following criteria: no injury = 0, injury to 25% of the field = 1, injury to 50% of the field = 2, injury to 75% of the field = 3, and diffuse injury = 4. The liver pathological injury was expressed as the sum of the individual score grades, 0 (no findings), 1 (mild), 2 (moderate), and 3 (severe), for each of the following six parameters: cytoplasmic color fading, vacuolization, nuclear condensation, nuclear fragmentation, nuclear fading, and erythrocyte stasis. Blind analysis was performed to determine the lesion degree of all samples.

### *In situ* Terminal Deoxynucleotidyl Transferase-Mediated Uridine Triphosphate Nick-End Labeling Assay

Apoptosis of pneumonocyte and hepatocytes were detected by transferase-mediated uridine triphosphate nick-end labeling (TUNEL) assay, which was performed according to the manufacturer's protocol (Roche, Switzerland).

### Flow Cytometry

For phenotype staining, cells were washed twice with PBS and were then stained with mouse antibodies of CD45, CD11c, F4/80, CD86, CD40, CD11b, Gr-1, Ly6G, and Ly6C for 30 min at 4°C according to the manufacturer's instructions. For intracellular staining, cells were incubated with monensin for 2 h and then washed twice with PBS. After resuspended with pulse vortex, cells were incubated with phenotype antibody for 30 min in dark at 4°C. After washing with PBS, 800 μl IC Fixation Buffer (eBioscience, USA) was added and incubated at 4°C for 30 min in the dark. After washed twice with 2 ml Permeabilization Buffer (eBioscience, USA) and centrifuged. Next, intracellular staining was performed in 100 μl of Permea-bilization Buffer by using TNF-α and IL-6 Ab. After incubating overnight in the dark at 4°C, cells were washed for twice with 2 ml Permeabilization Buffer. Cells were analyzed by FACS Calibur (Becton Dickinson, USA). An isotype control was used for each antibody.

### PI Uptake Assay

Collected cells were washed twice with PBS, and then, they were incubated with PI dye solution (2 μg/ml, Beyotime) for 15 min at RT. The PI uptake by cells was analyzed by FACS Calibur (Becton Dickinson).

### Enzyme-Linked Immunosorbent Assay

The concentrations of TNF-α, IL-12, and IL-6 in serum and in culture supernatant of BMDMs and peritoneal macrophages were determined by the mouse enzyme-linked immunosorbent assay (ELISA) kit (Biolegend, USA) according to the standard procedure, respectively. In brief, each well of the plate was coated with capture antibody and incubated overnight at 4°C. The next day, the plate were washed four times by phosphate buffered saline (PBS, containing 0.1% Tween 20) and were blocked with 200 μl Assay Diluent (1% BSA) at 37°C for 1 h with shaking at ~500 rpm. Following, the wash step should be performed similarly. Then, each well was incubated with 100 μl sample at 37°C for 2 h with shaking. After washing for four times, we added 100 μl biotinylated antibodies to each well and incubated at 37°C for 1 h. After washing for four times, we added 100 μl streptavidin-HRP to each well and incubated at 37°C for 30 min. After washing for five times, 100 μl TMB was added to each well and incubated at 37°C. After appropriate time, 100 μl stop solution was added to each well and the plates were read at 450 nm using a microplate reader (BioTek). All samples were assayed in duplicates.

### Immunoblotting Analysis

Proteins extracted from the corresponding cells resolved using 10% SDS-PAGE. And then, the proteins were blotted onto membranes (Millipore) in a transfer solution at 100 V for 1 h. After being blocked with 3% BSA, the membranes were incubated with primary antibody for p-RIP1(1:1000, CST, USA), RIP3 (1:1000, CST, USA), p-RIP3 (1:1000, CST, USA), p38 (1:1000, CST, USA), p-p38 (1:1000, CST, USA), p65 (1:1000, CST, USA), p-p65 (1:1000, CST, USA), JNK (1:1000, CST, USA), p-JNK (1:1000, CST, USA), ERK1/2 (1:1000, CST, USA), and p-ERK1/2 (1:1000, CST, USA), and with β-actin antibody (1:1000, CST, USA) at 4°C overnight. Next day, after washing, the membranes were incubated with secondary antibody HRP-labeled Goat Anti-Rabbit (1:3000, Beyotime Biotechnology, China) or HRP labeled Rabbit Anti-Mouse (1:3000, Beyotime Biotechnology, China). In the end, the membranes were detected with ECL kit (Thermo, USA) with chemiluminescence. β-actin was used as an internal control.

### Statistical Analysis

All experiments were performed at least three times. The data were presented as the mean ± standard error of the means. Data were analyzed using one-way ANOVA, Student's *t*-test or two-way ANOVA. Survival curves were estimated by using Kaplan–Meier method and the Log-rank test was applied to determine the differences of survival rate. Statistically significant was assumed for *P* < 0.05 (^*^), *P* < 0.01 (^**^), *P* < 0.001 (^***^), and not significant (ns).

## Results

### Expression of Necroptosis-Related Molecules in Mice Challenged With LPS

**Figure 1:**
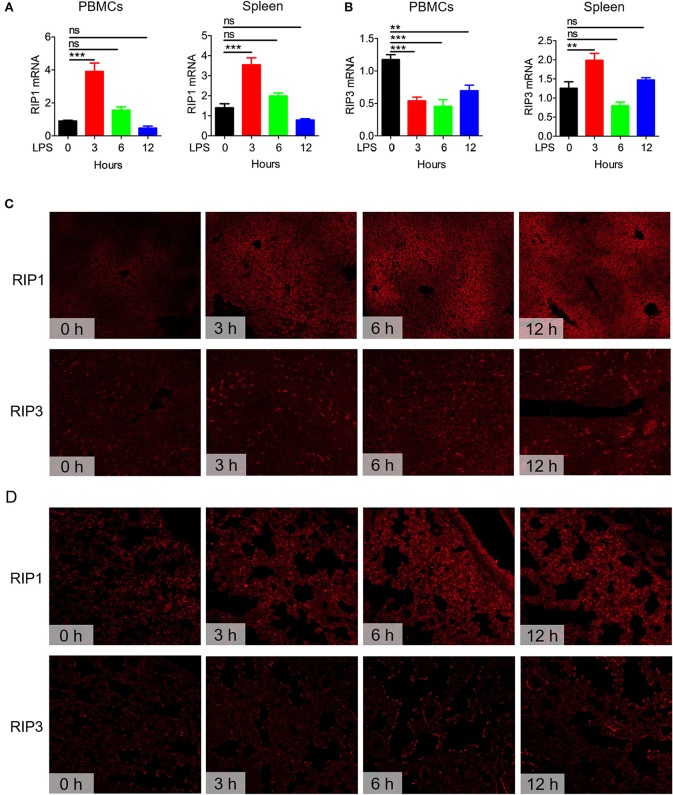
Expression of the necroptosis-related genes RIP1 and RIP3 in mice undergoing endotoxin shock. C57BL/6 mice were challenged with lipopolysaccharide (LPS; 10 μg/g body weight) for 0, 3, 6, or 12 h (*n* = 8). The expression levels of RIP1 and RIP3 in peripheral blood mononuclear cells (PBMCs) and spleen tissues were examined. **(A,B)** Q-PCR analysis of RIP1 and RIP3 mRNA transcript expression levels in PBMCs and spleen tissues. Data are presented as means ± S.E.M. of triplicates. **(C,D)** Immunofluorescence staining to detect RIP1 and RIP3 protein expression in liver and lung tissue sections. Red represents RIP1 or RIP3. Data shown are representative of three independent experiments. Error bars represent S.E.M.; ^**^p < 0.01, ^***^p < 0.001, as determined by ANOVA test; ^ns^*p* > 0.05.

Necroptosis is known to play an important role in the pathogenesis of many inflammatory diseases (17, 35, 36). RIP1, RIP3, and MLKL are critical regulators of necroptosis (37). Before exploring the effects of zVAD on the pathogenesis of endotoxin shock, we first examined the expression levels of these necroptosis-related genes in mice challenged with LPS. We found that the mRNA transcript levels of RIP1, RIP3, and MLKL significantly increased in PBMCs and spleens from mice challenged with LPS (Figures 1A,B and Figure S1). Moreover, we measured levels of these proteins in liver and lung tissues, and found that RIP1 and RIP3 were both significantly increased in livers and lungs (Figures 1C,D). Overall, these data suggested that necroptosis-related molecules are upregulated in mice undergoing endotoxin shock and necroptosis may contribute to the pathogenesis of endotoxic shock.

### Intraperitoneal Injection of zVAD Reduces Mortality in LPS-Challenged Mice

**Figure 2:**
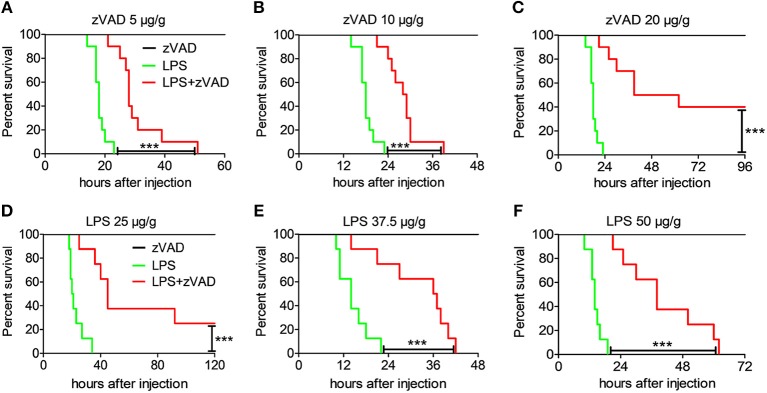
Intraperitoneal injection of zVAD results in reduced mortality of mice challenged with LPS. **(A–C)** Groups of C57BL/6 mice were pretreated with various doses of zVAD (5, 10, or 20 μg/g body weight) or vehicle (saline) for 2 h followed by lipopolysaccharide (LPS) challenge (40 μg/g body weight), and mortality was observed (*n* = 10 mice/group). The Kaplan–Meier method was used to estimate overall survival and survival rates were determined using the Log-rank test. **(D–F)** Groups of C57BL/6 mice were pretreated with zVAD (20 μg/g body weight) or vehicle (saline) followed by LPS challenge (25, 37.5, or 50 μg/g body weight), and mortality was observed (*n* = 8 mice/group). The Kaplan–Meier method was used to estimate overall survival and survival rates were determined using the Log-rank test; ^***^P < 0.001.

Although many studies have shown that necroptosis plays a critical role in the pathogenesis of inflammatory diseases, there is still a lot of controversy about the effect of zVAD (24, 25). zVAD, a pan-caspase inhibitor, can induce necroptosis after stimulation with LPS in a mouse model of endotoxin shock (38). However, the underlying mechanism of zVAD, remain poorly understood. To understand the role of zVAD in the pathogenesis of endotoxin shock, mice were pretreated with various doses of zVAD before challenge with LPS and mouse mortality and survival time were recorded. Intriguingly, treatment with zVAD could significantly extend mice survival by hours and increased the survival rate (Figures 2A–C). Additionally, mice were administered zVAD at a dose of 20 μg/g or not, followed by stimulation with different doses of LPS and the observation of mouse mortality. As expected, treatment with zVAD significantly reduced the mortality of mice treated with various doses of LPS (Figures 2D–F). These data establish that zVAD can indeed reduce LPS-induced mortality in mice.

### Intraperitoneal Injection of zVAD Attenuated LPS-Induced Lung and Liver Injury and Inhibited LPS-Induced Inflammation

**Figure 3:**
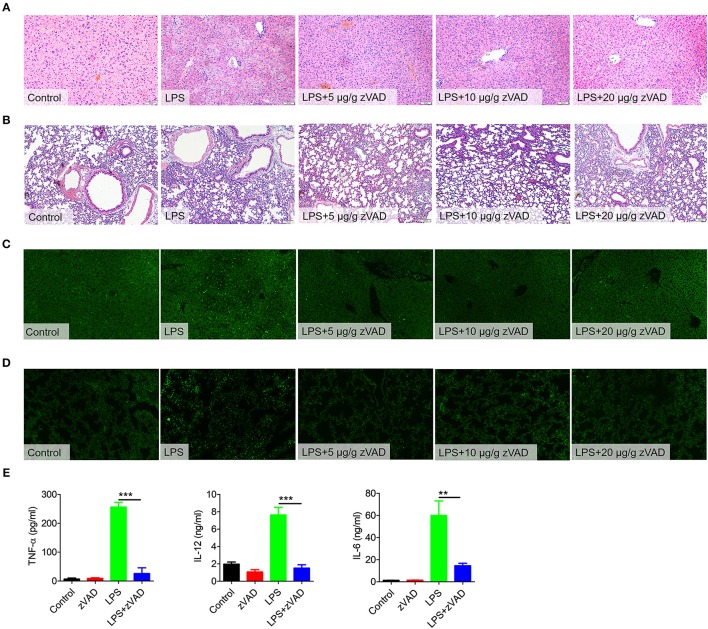
Intraperitoneal injection of zVAD ameliorated pathology and inflammatory cytokine secretion in mice challenged with lipopolysaccharide (LPS). **(A–D)** C57BL/6 mice were pretreated with different doses of zVAD (5, 10, or 20 μg/g body weight) or vehicle (saline) prior to LPS challenge (10 μg/g body weight). After 12 h, liver and lung tissues were collected. **(A,B)** Paraffin-embedded liver **(A)** and lung **(B)** sections were stained with hematoxylin and eosin. **(C,D)** Apoptotic cells in liver **(C)** and lung **(D)** tissues were detected by TUNEL. **(E)** C57BL/6 mice were pretreated with zVAD (20 μg/g body weight) or vehicle (saline) followed by LPS challenge (10 μg/g body weight). After 6 h, levels of TNF-α, IL-12, and IL-6 in serum were measured by ELISA. Data are presented as means ± S.E.M. of triplicate measurements and are representative of three independent experiments. Error bars represent S.E.M.; ^**^p < 0.01, ^***^p < 0.001, as determined by ANOVA test.

After establishing that zVAD can reduce the mortality of mice undergoing endotoxin shock, we next assessed the effects of zVAD on LPS-induced pathology. Mice were pretreated with various doses of zVAD for 2 h followed by LPS challenge for 12 h and then pathological lesions were observed. Treatment with zVAD alone had no obvious effect on histological assessments, TUNEL staining or MPO activity of mouse tissues (Figures S2, S10). In contrast to mice treated with LPS and zVAD, obvious liver and lung pathology, such as hepatocyte karyolysis, karyopyknosis, inflammatory cell infiltration and disordered hepatic cord arrangement, could be observed in the LPS-challenged group (Figures 3A,B). Furthermore, the MPO activity also indicated that the infiltration of neutrophils in liver and lung were decreased after treated with zVAD (Figure S10). Moreover, TUNEL staining revealed that the percentage of apoptotic cells in lung and liver tissues from LPS and zVAD treated mice was significantly reduced compared to that from LPS-treated mice (Figures 3C,D). As is known, overexpression of pro-inflammatory cytokines is a major driver of inflammatory responses and tissue injuries in endotoxin shock. Thus, we measured levels of pro-inflammatory cytokines in serum from endotoxin shock mice treated with LPS and zVAD or LPS alone. Similarly, treatment with LPS and zVAD markedly reduced the concentrations of TNF-α, IL-12, and IL-6 in endotoxin shock mice (Figure 3E). Furthermore, we build a post-treating model. The mice were challenged by LPS for 1 h followed by zVAD treatment. As shown in Figure S9, post-treatment of zVAD could not alleviate the condition of LPS-induced endotoxin shock model, even that post-treatment of zVAD could seemly increase the inflammation cytokines in LPS-challenged mice. Based on these data, we demonstrated that zVAD could ameliorate LPS-induced lung and liver injuries and inhibit LPS-induced pro-inflammatory responses.

### No Effect of Intravenously Injected zVAD on LPS-Induced Endotoxic Shock Pathology and Survival

**Figure 4:**
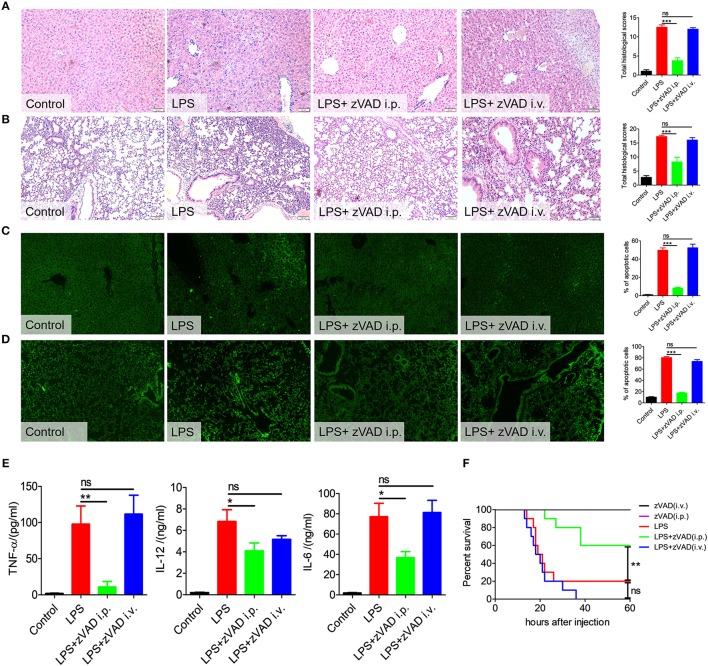
Intravenous injection of zVAD showed no effect on pathology, survival or inflammatory cytokine secretion in mice challenged with lipopolysaccharide (LPS). C57BL/6 mice were injected with zVAD (20 μg/g body weight) by intraperitoneal or intravenous injection followed by LPS challenge (10 μg/g body weight). **(A–D)** After 12 h, liver and lung tissues were collected. **(A,B)** Paraffin-embedded liver **(A)** and lung **(B)** tissue sections were stained with hematoxylin and eosin. **(C,D)** Apoptotic cells in liver **(C)** and lung **(D)** tissues were detected by TUNEL. **(E)** After 6 h, levels of TNF-α, IL-12, and IL-6 in serum were measured by ELISA. Data are presented as means ± S.E.M. of triplicate measurements. **(F)** C57BL/6 mice were injected with zVAD (20 μg/g body weight) by intraperitoneal or intravenous injection followed by LPS challenge (25 μg/g body weight). The mortality was observed (*n* = 10 mice/group). The Kaplan–Meier method was used to estimate overall survival and survival rates were determined using the Log-rank test. Data shown are representative of three independent experiments. Error bars represent S.E.M.; ^*^p < 0.05, ^**^p < 0.01, ^***^p < 0.001, as determined by ANOVA test; ^ns^*p* > 0.05.

Macrophages in the abdominal cavity play critical roles in the pathogenesis of endotoxin shock (2–4). Considering the above results, we proposed that the intraperitoneal injection of zVAD may ameliorate LPS-induced endotoxic shock by inducing the necroptosis of macrophages in the abdominal cavity. To test this hypothesis, we explored the effect of intravenously injected zVAD on LPS-induced endotoxic shock. Mice were injected with zVAD either intravenously or intraperitoneally and then 2 h later were challenged with LPS. Interestingly, intraperitoneal (i.p.) injection of zVAD reduced mortality, alleviated tissue pathology and decreased the percentage of apoptotic cells in the lung and liver, whereas intravenous (i.v.) injection of zVAD showed no significant effect (Figures 4A–D,F). The same trend occurred for levels of TNF-α, IL-12, and IL-6 (Figure 4E). Together with above data, these findings suggest that intraperitoneal injection of zVAD could ameliorate LPS-induced endotoxic shock, whereas intravenous injection of zVAD showed no impact.

### Intraperitoneal Injection of zVAD Alleviated LPS-Induced Endotoxic Shock by Inducing the Necroptosis of Macrophages in the Abdominal Cavity

**Figure 5:**
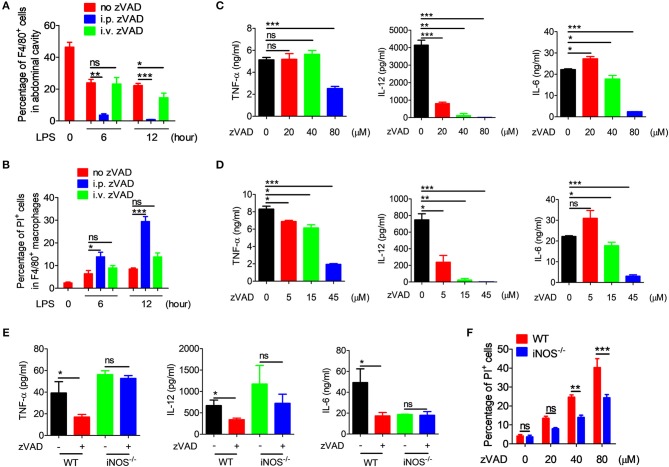
zVAD induced macrophage necroptosis and blocked the secretion of proinflammatory cytokines. **(A,B)** After intraperitoneal or intravenous injection of zVAD (20 μg/g of body weight) or vehicle (saline) for 2 h, mice were challenged with lipopolysaccharide (LPS; 10 μg/g body weight) for 6 or 12 h. The percentages of F4/80^+^ cells **(A)** and PI uptake of F4/80^+^ cells **(B)** were measured by flow cytometry. **(C,D)** Bone marrow-derived macrophages (BMDMs) or peritoneal macrophages induced by thioglycollate medium were pretreated with zVAD as indicated or with vehicle (PBS) for 30 min followed by LPS administration (100 ng/ml) for 24 h. Levels of secreted TNF-α, IL-12, and IL-6 in culture supernatants of BMDMs **(C)** or peritoneal macrophages **(D)** were analyzed by ELISA. **(E)** C57BL/6 and iNOS^−/−^ mice were pretreated with zVAD (20 μg/g body weight) or vehicle (saline) for 2 h followed by LPS challenge (10 μg/g) for 6 h. Levels of TNF-α, IL-12, and IL-6 in serum were measured by ELISA. **(F)** BMDMs generated from C57BL/6 and iNOS^−/−^ mice were pretreated with or without zVAD (20, 40, or 80 μM) for 30 min followed by LPS stimulation (100 ng/ml). PI uptake by BMDMs was assessed by flow cytometry at 24 h. Data shown are representative of three independent experiments. Error bars represent S.E.M.; ^*^p < 0.05, ^**^p < 0.01, ^***^p < 0.001, as determined by ANOVA test; ^ns^*p* > 0.05.

As is known, macrophages play a key role in the pathogenesis of endotoxic shock by producing large amounts of inflammatory cytokines and NO (6–8). We guess that the different treatment effect of i.p. and i.v. administration on endotoxin shock may be related to macrophages within the abdominal cavity. To explore whether the intraperitoneal injection of zVAD alleviates LPS-induced endotoxic shock by inducing the necroptosis of macrophages in the abdominal cavity, *in vivo* studies were designed to examine the necroptosis of peritoneal macrophages from endotoxin shock mice treated with zVAD either intraperitoneally or intravenously. Mice were pretreated with zVAD i.p. or i.v. for 2 h, followed by LPS administration for 6 or 12 h and then peritoneal cells were collected. The percentage of F4/80^+^ macrophages and uptake of PI in F4/80^+^ macrophages were examined. As shown in Figure 5A, compared with LPS-treated mice, intraperitoneal injection of zVAD significantly reduced the percentage of F4/80^+^ macrophages in the abdominal cavity, while intravenous injection of zVAD showed no significant effect. The PI uptake assay showed that, compared with LPS-treated mice, the uptake of PI in F4/80^+^ macrophages from the abdominal cavity of mice treated with intraperitoneal injection of zVAD prior to LPS challenge was significantly higher, while there was no difference between the LPS-treated mice and mice treated with intravenous injection of zVAD prior to LPS challenge (Figure 5B). What's more, we also found that intraperitoneal injection of zVAD alone showed no effect on the percentage of F4/80^+^ macrophages in the abdominal cavity and the uptake of PI in F4/80^+^ macrophages (Figure S4). In addition, peritoneal macrophages were collected. After treated with zVAD or PBS, cells were stimulated with LPS. After 24 h, proteins were collected and used to detect the expression of p-RIP1 (representational necroptosis protein). As shown in Figure S5, the level of p-RIP1 was increased markedly. To sum up, these data suggest that the intraperitoneal injection of zVAD can indeed induce macrophage necroptosis in the abdominal cavity.

To further confirm whether or not the zVAD-induced reduction in inflammatory cytokines in endotoxin shock mice was a consequence of the necroptosis of macrophages, BMDMs and peritoneal macrophages were pretreated with different doses of zVAD for 30 min followed by LPS stimulation for 24 or 48 h, followed by the measurement of levels of TNF-α, IL-12, and IL-6 in the culture supernatant. We found that at 24 and 48 h, pre-treatment with zVAD could significantly reduce levels of TNF-α, IL-12, and IL-6 induced by LPS (Figures 5C,D and Figure S3), suggesting that zVAD reduced the secretion of proinflammatory cytokines by LPS-activated macrophages mainly through the induction of macrophage necroptosis.

To investigate in more detail the underlying mechanism involved in regulating the necroptosis of macrophages induced by LPS plus zVAD treatment, we focused on iNOS. It is known that iNOS is induced after activation by endotoxins to generate NO, which functions as host defense molecule that protects against invading micro-organisms (37, 39). As in previous study from our laboratory, NO exerted robust effects on the polarization of macrophages (28). To investigate whether NO is involved in the activity of zVAD, iNOS^−/−^, and WT mice were treated with zVAD for 2 h followed by LPS challenge and then serum was collected to measure levels of TNF-α, IL-12, and IL-6. Furthermore, at 12 h liver and lung tissues were collected for hematoxylin and eosin staining. Analysis of the tissues clearly demonstrated that treatment with zVAD could significantly inhibit the release of TNF-α, IL-12, and IL-6 in mice undergoing endotoxin shock, whereas treatment with zVAD alone showed no effect on the release of TNF-α, IL-12, or IL-6 in iNOS^−/−^ mice undergoing endotoxin shock (Figure 5E). Similar results were observed for liver and lung tissue pathology (Figure S6). Moreover, we also examined the effect of iNOS on the necroptosis of BMDMs induced by LPS and zVAD and found that BMDMs from iNOS^−/−^ mice showed lower levels of PI uptake compared with those from WT mice (Figure 5F). Additionally, LPS plus zVAD induced phosphorylation of RIP3 was also examined by western blotting. BMDMs from iNOS^−/−^ mice showed lower levels of phosphorylation of RIP3 compared with those from WT mice (Figure S7), suggesting that iNOS promotes the necroptosis of macrophages treated with LPS and zVAD. Taken together, we can conclude that the intraperitoneal injection of zVAD can alleviate LPS-induced endotoxic shock by inducing the necroptosis of macrophages in a process that involves NO.

### Intraperitoneal Injection of zVAD Alleviated LPS-Induced Pro-inflammatory Responses in Macrophages From Mice Undergoing Endotoxic Shock by Promoting MDSC Accumulation

**Figure 6:**
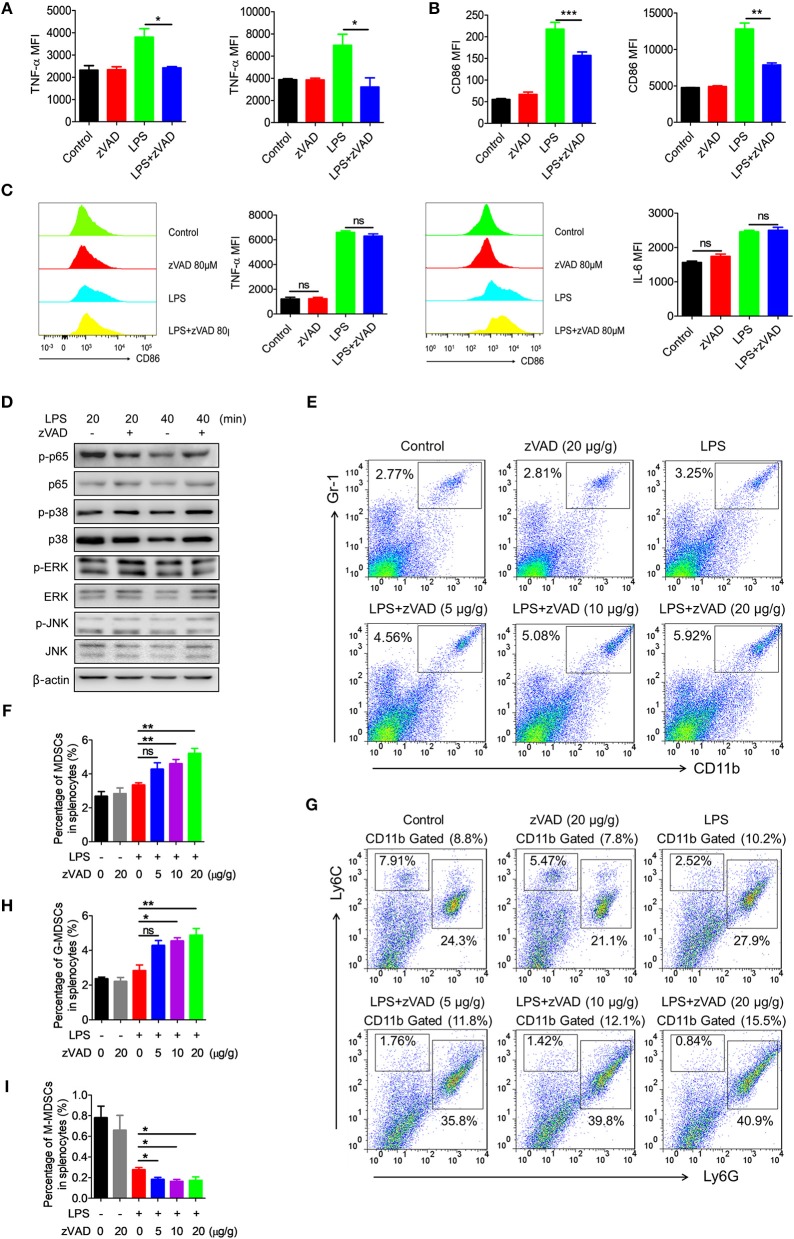
Intraperitoneal injection of zVAD contributes to the accumulation of myeloid-derived suppressor cells (MDSCs) in mice undergoing endotoxic shock. **(A,B)** After intraperitoneal injection of zVAD (20 μg/g body weight) or vehicle (saline) for 2 h, mice were challenged with lipopolysaccharide (LPS; 10 μg/g body weight) or not for 12 h. The expression levels of CD86 and TNF-α in F4/80^+^ cells in spleens and livers were measured by flow cytometry. **(C,D)** Bone marrow-derived macrophages (BMDMs) generated from C57BL/6 mice were pretreated with zVAD (80 μM) for 30 min or not. **(C)** Cells were then stimulated with LPS for 24 h. The cells were collected to detect the concentration of intracellular IL-6 and TNF-α. **(D)** Cells were then stimulated with LPS for 20 or 40 min. Cell lysates were prepared and subjected to immunoblotting with the indicated antibodies. **(E–I)** C57BL/6 mice were pretreated with different doses of zVAD (5, 10, or 20 μg/g body weight) or vehicle (saline) for 2 h followed by LPS challenge (10 μg/g body weight) for 12 h. Mice were then challenged with LPS (10 μg/g body weight) or not for 12 h. The percentages of MDSCs **(E,F)**, G-MDSCs **(G,H)**, and M-MDSCs **(G,I)** in spleens were determined using flow cytometry. Data shown are representative of three independent experiments. Error bars represent S.E.M.; ^*^p < 0.05, ^**^p < 0.01, ^***^p < 0.001, as determined by ANOVA test; ^ns^*p* > 0.05.

**Figure 7:**
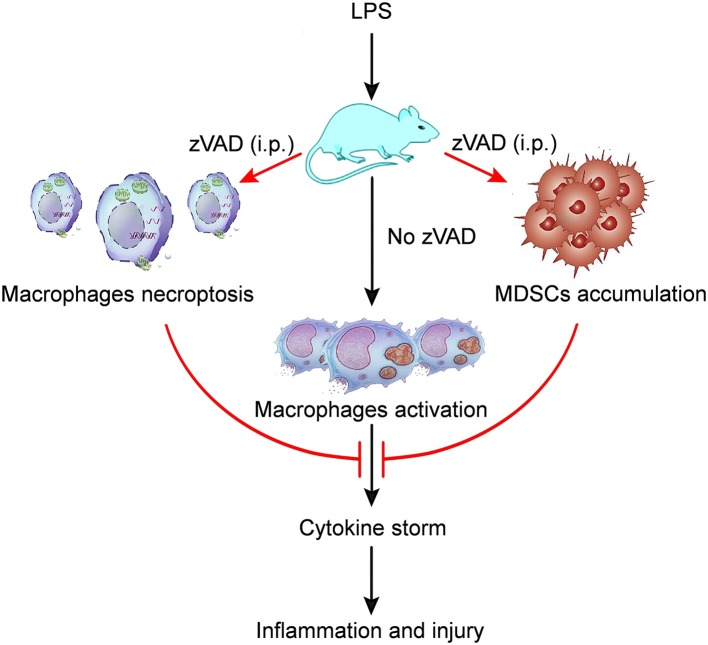
A model of the mechanism whereby zVAD ameliorates endotoxin shock pathology. The schematic diagram indicates that mice treated with lipopolysaccharide (LPS) would undergo macrophage activation, leading to cytokine storm that would cause inflammation and injury in mice. However, mice pretreated with zVAD (i.p.) prior to LPS challenge would exhibit macrophage necroptosis and the accumulation of MDSCs. Both of the above effects can largely inhibit macrophage activation, thereby reducing cytokine production and avoiding severe damage. Thus, zVAD can alleviate LPS-induced endotoxic shock by inducing the necroptosis of macrophages and promoting MDSCs-mediated inhibition of macrophage activation.

Because the intraperitoneal injection of zVAD can alleviate LPS-induced endotoxic shock and reduce levels of proinflammatory cytokines, we asked whether the intraperitoneal injection of zVAD could alleviate LPS-induced proinflammatory responses in macrophages *in vivo*. Thus, the activation of macrophages in spleens and livers from endotoxin shock mice treated with zVAD or vehicle (saline) was assessed. As shown in Figures 6A,B treatment with zVAD (i.p.) could significantly block the secretion of TNF-α and inhibit LPS-induced CD86 expression on macrophages in spleens and livers. However, considering that zVAD could not affect LPS-induced production of IL-6 and TNF-α, and activation of MAPKs and NF-κB signaling pathways in BMDMs (Figures 6C,D), we found that zVAD could not directly affect LPS-induced proinflammatory responses in macrophages. Notably, studies from our group and others have shown that MDSCs play a protective role in the pathogenesis of endotoxin shock by inhibiting proinflammatory responses in macrophages (40, 41). Therefore, it is conceivable that zVAD may promote the accumulation of MDSCs that then inhibit LPS-induced proinflammatory responses in macrophages (Figure S8). Accordingly, the percentages of MDSCs in spleens from endotoxin shock mice treated with or without zVAD were measured and we found that zVAD markedly promoted the accumulation of MDSCs in endotoxin shock mice (Figures 6E–H). We found that the proportion of MDSCs and G-MDSCs increased in a concentration-dependent manner, while the proportion of M-MDSCs decreased (Figures 6G,I). Together, these data demonstrated that the intraperitoneal injection of zVAD promotes the accumulation of MDSCs that can inhibit LPS-induced pro-inflammatory responses in macrophages *in vivo*. In summary, our results supported our hypothesis that the intraperitoneal injection of zVAD alleviated LPS-induced endotoxic shock by inducing the necroptosis of peritoneal macrophages and promoting MDSC-mediated inhibition of macrophage activation (Figure 7).

## Discussion

The role of necroptosis in the occurrence and development of inflammation remains the subject of debate. Although most studies have shown that necroptosis promotes inflammation, other studies have shown that it can alleviate inflammation (42). According to our recent research, intraperitoneal injection of zVAD can significantly ameliorate endotoxic shock. Further analysis has found that zVAD can cause macrophage necroptosis and block the secretion of inflammatory cytokines, while also inhibiting the polarization of M1 macrophages by promoting MDSCs aggregation, which in turn can inhibit the secretion of inflammatory cytokines.

We also measured the neutrophil infiltration in liver and lung by MPO activity detection kit. But as is reported previously, inhibition of apoptosis prevents extravasation of neutrophils but dose not effect overall recruitment in a sepsis model. While extravasation is critical for PMN mediated tissue injury and may effect relative MPO activity as the PMNs may remain inactive (43). Therefore, the extravasation of neutrophil should be determined in the future study.

Interestingly, we found in this study that the intraperitoneal injection of zVAD can significantly ameliorate endotoxin shock, whereas its intravenous injection cannot. A major difference between consequences of the two injection methods is the necroptosis of peritoneal macrophages. Intraperitoneal injection of zVAD can cause necroptosis of macrophages and block inflammatory cytokine secretion in LPS challenged mice, whereas the intravenous injection of zVAD shows no effect. After LPS stimulation, peritoneal macrophages secrete various inflammatory cytokines and aggravate endotoxic shock.

In addition, we have also found that the intraperitoneal injection of zVAD can promote the aggregation of MDSCs in LPS challenged mice, thereby inhibiting the polarization of M1 macrophages, reducing inflammatory cytokine secretion, and ameliorating disease. However, intravenous injection of zVAD does not promote the aggregation of MDSCs in LPS challenged mice, which suggests that zVAD itself may not affect MDSC aggregation. Therefore, we hypothesize that some nucleic acids or protein substances released after the necroptosis of macrophages may regulate the aggregation of MDSCs in LPS challenged mice.

The main research agent that we used in this study was zVAD. To be more certain that the effects that we observed *in vivo* were indeed dependent upon necroptosis, knockout mice lacking RIP3 should be used in future studies to confirm that zVAD exerts a therapeutic effect by causing the necroptosis of macrophages.

Studies have reported that NO can regulate both apoptosis and necroptosis (44, 45). Our previous study found that autocrine NO produced by macrophages can inhibit the polarization of macrophages to M1 cells (28). In conclusion, all of this data indicates that NO has an important regulatory effect on the survival and activation of immune cells. However, to fully elucidate the mechanism, further research will be required.

In summary, our research demonstrated that zVAD can induce the necroptosis of peritoneal macrophages and promote the aggregation of MDSCs in LPS-induced endotoxic shock. Moreover, NO may play a role in zVAD-dependent amelioration of endotoxic shock. This finding will help us to understand the underlying mechanism and perhaps find novel therapies for the treatment of endotoxic shock. However, further investigations aimed at elucidating the underlying mechanisms are still required.

## Data Availability

The raw data supporting the conclusions of this manuscript will be made available by the authors, without undue reservation, to any qualified researcher.

## Ethics Statement

This study was carried out in accordance with the recommendations of Guide for the Care and Use of Laboratory Animals of Jining Medical University and Animal Care Committee at Jining Medical University. The protocol was approved by the Animal Care Committee at Jining Medical University. All procedures were performed under sodium pentobarbital anesthesia, and all efforts were made to minimize suffering of the animals.

## Author Contributions

HX, GD, and XL designed and supervised the study. XL, XY, YZ, HZ, QM, YY, JZ, HS, ZN, ZL, CL, HW, FS, and GD performed the experiments. FY, JD, YX, XM, FS, and GD analyzed the data and wrote the paper.

### Conflict of Interest Statement

The authors declare that the research was conducted in the absence of any commercial or financial relationships that could be construed as a potential conflict of interest.
