# Supplementary material for: The Caspase Inhibitor Z-VAD-FMK Alleviates Endotoxic Shock via Inducing Macrophages Necroptosis and Promoting MDSCs-Mediated Inhibition of Macrophages Activation
Source: Front Immunol. 2019 Aug 2;10:1824. doi: 10.3389/fimmu.2019.01824 (PMC6687755; doi:10.3389/fimmu.2019.01824)
Supplement: Supplementary file 1 [file Data_Sheet_1.PDF]

Supplementary Data

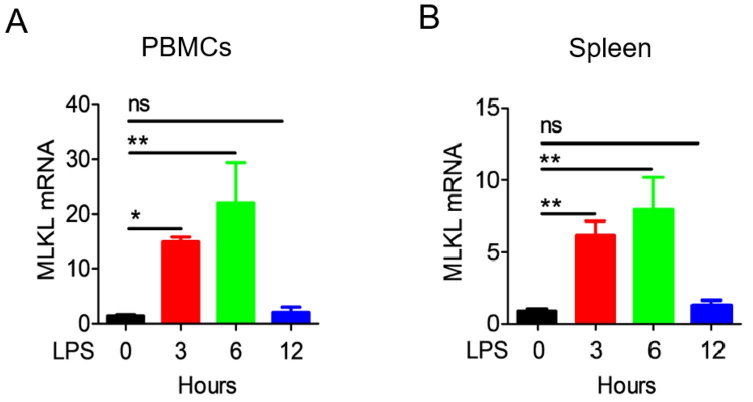

Figure S1 Expression of MLKL in mice challenged with LPS.

C57BL/6 mice were challenged with LPS for 0, 3, 6 and 12 hours. The expressions of MLKL in PBMCs (A) and spleen (B) were measured by Q-PCR. Data shown are representative of three independent experiments. Error bars represent S.E.M. *p < 0.05, **p < 0.01, ***p < 0.001, as determined by ANOVA test; ns denotes p > 0.05.

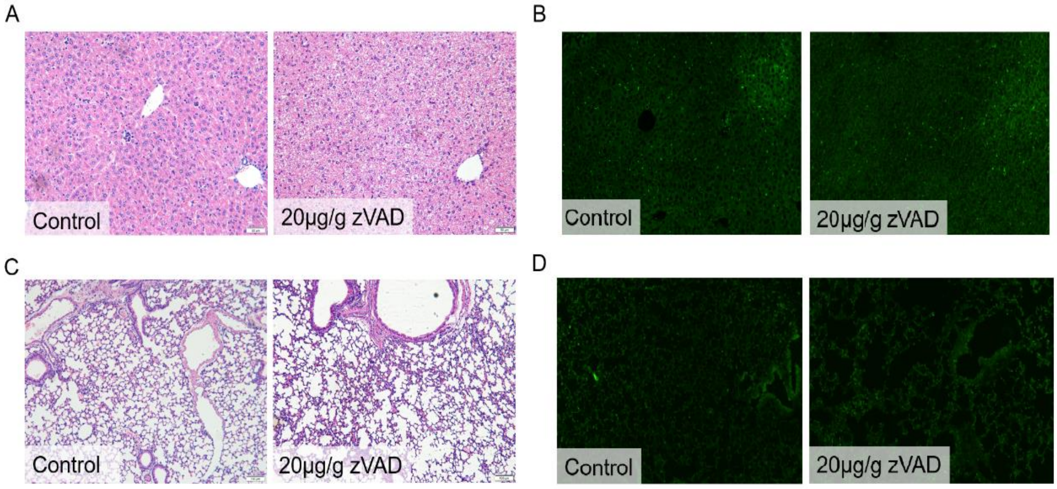

Figure S2 zVAD alone could not affect the liver and lung of mice.

C57BL/6 mice were injected with zVAD (20 μg/g of body weight) or vehicle through the tail vein for 12 hours, and then, the lungs and livers were fixed in 4% paraformaldehyde and embedded with paraffin. The liver (A) and lung (C) were stained with hematoxylin and eosin. The apoptotic cells in liver (B) and lung (D) were

detected by TUNEL assay. Data shown are representative of three independent experiments.

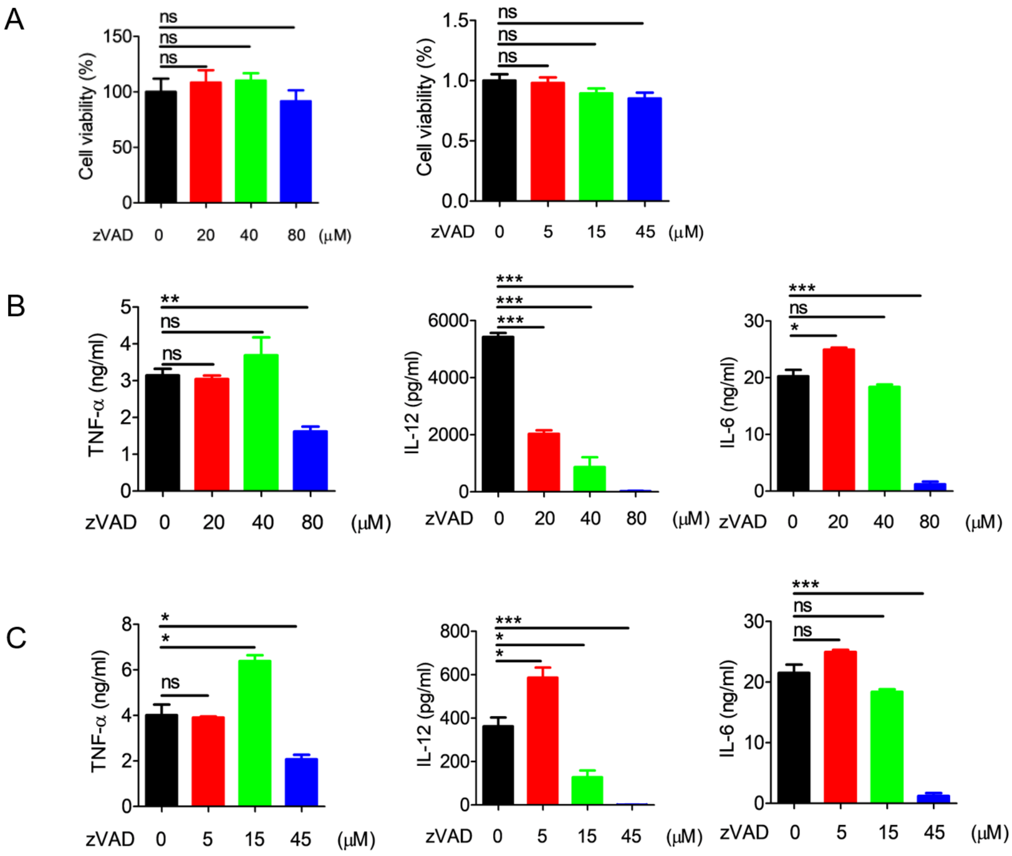

Figure S3 zVAD blocked LPS-induced secretions of TNF-α, IL-12 and IL-6 in BMDMs and peritoneal macrophages.

BMDMs and peritoneal macrophages generated from C57BL/6 mice were pretreated with different doses of zVAD as indicated in figure legend followed by LPS stimulation (100 ng/ml) respectively or not. At 48h, the absorbance at 450 nm was measured in a microplate reader (A). And the levels of TNF-α, IL-12 and IL-6 in culture supernatant of BMDMs (B) and peritoneal macrophages (C) were detected by ELISA. The data are presented as the mean ± SEM of triplicates. Error bars represent S.E.M. *$p < 0.05$, **$p < 0.01$, ***$p < 0.001$, as determined by ANOVA test; ns denotes $p > 0.05$.

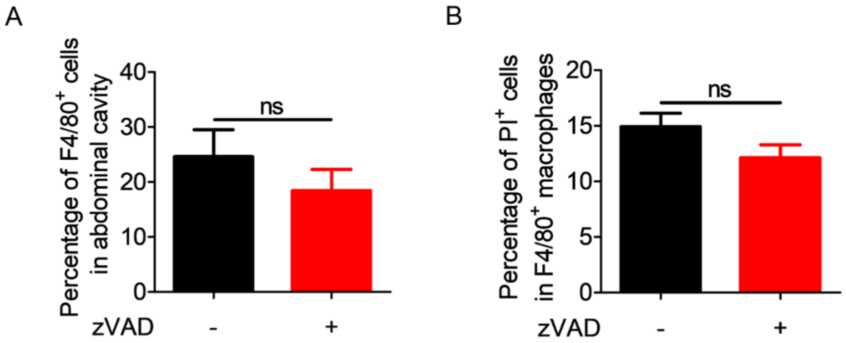

Figure S4 zVAD alone did no significant effect on the percentage of F4/80$^+$ macrophages in the abdominal cavity and uptake of PI in F4/80$^+$ macrophages

C57BL/6 mice were injected with zVAD (20 μg/g of body weight) or vehicle through abdominal cavity for 12 hours, and then, the peritoneal cells were measured by flow cytometry. The data are presented as the mean ± SEM of triplicates. Error bars represent S.E.M, as determined by Student's t-test; ns denotes $p > 0.05$;

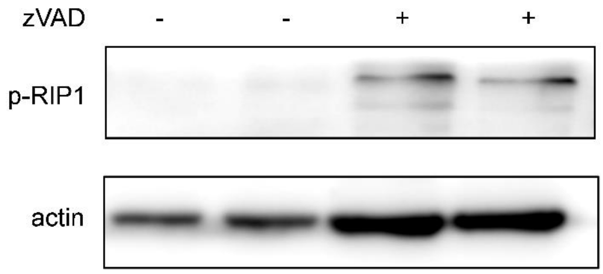

Figure S5 The peritoneal macrophages undergo necroptosis when treated with LPS plus zVAD.

Peritoneal macrophages generated from C57BL/6 mice were pretreated with zVAD (45μM) or PBS followed by LPS stimulation (100 ng/ml). Proteins extracted from the peritoneal macrophages were used for immunoblotting analysis.

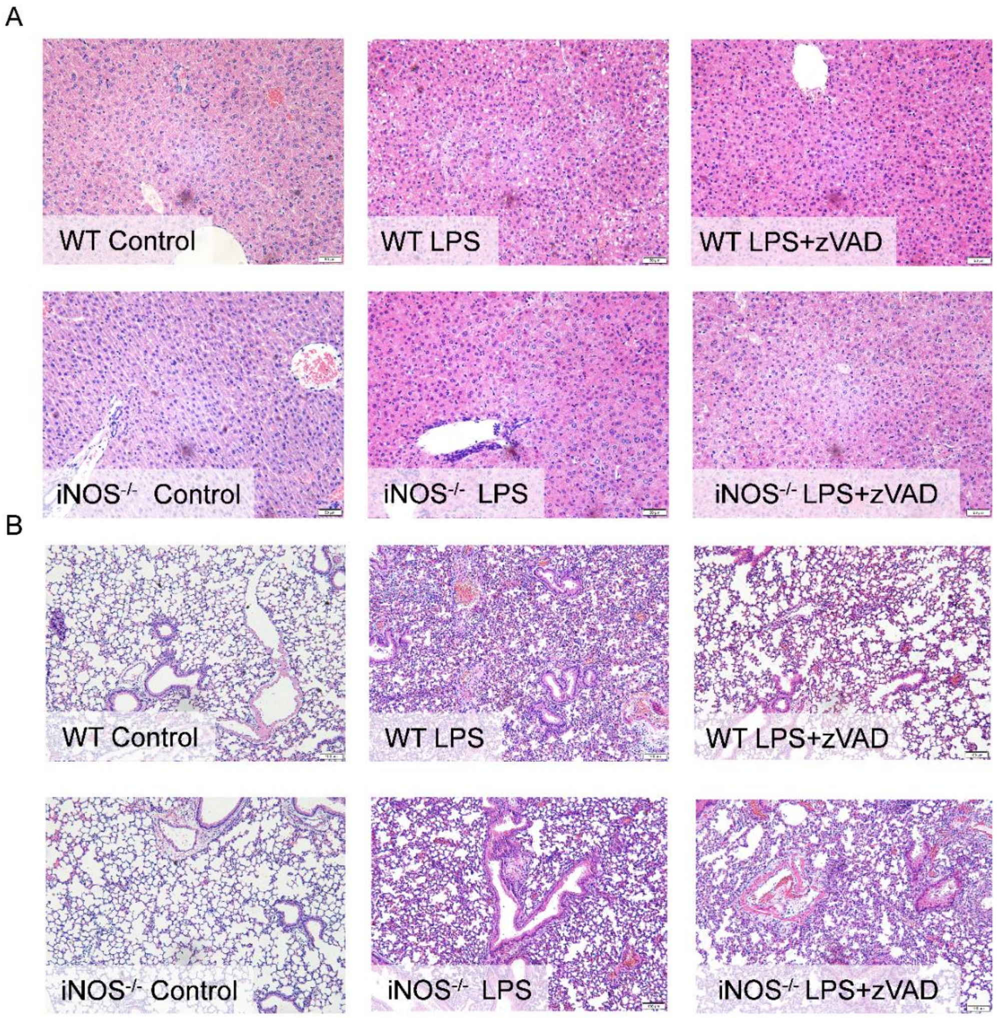

Figure S6 iNOS deficiency offset the treatment of zVAD on the pathological damage of mice with endotoxin shock.

C57BL/6 and iNOS$^{-/-}$ mice were injected with zVAD (20 μg/g of body weight) or vehicle through abdominal cavity prior to LPS challenge for 12 hours, and then, the lungs and livers were fixed in 4% paraformaldehyde and embedded with paraffin. The livers (A) and lungs (B) were stained with hematoxylin and eosin. Data shown are representative of three independent experiments.

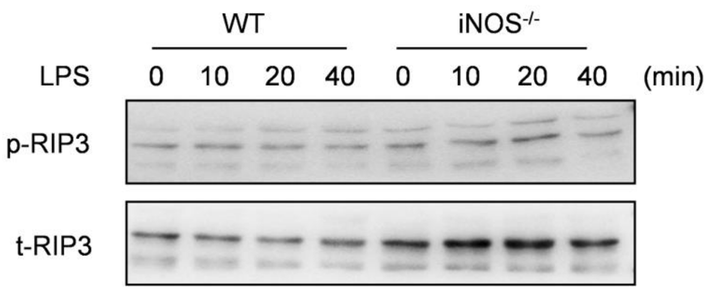

Figure S7 BMDMs from iNOS$^{-/-}$ mice showed lower levels of phosphorylation of RIP3 compared with those from WT mice

BMDMs generated from C57BL/6 and iNOS$^{-/-}$ mice were pretreated with zVAD (20μM) followed by LPS stimulation (100 ng/ml) for different time intervals respectively. Proteins extracted from the BMDMs were used for immunoblotting analysis.

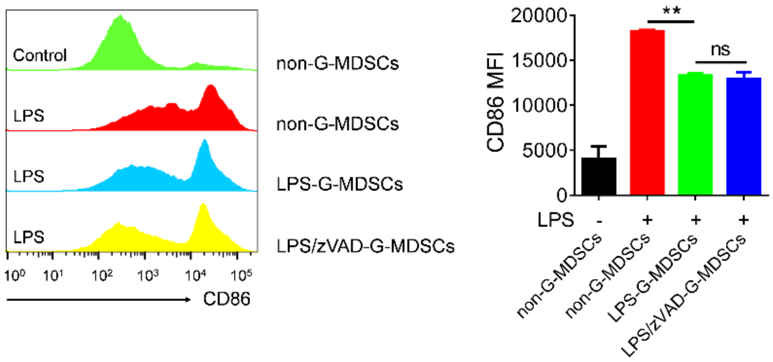

Figure S8 MDSCs can inhibit the activity of BMDMs while there is no significant difference between the LPS-G-MDSCs and the LPS/zVAD-G-MDSCs group.

Spleen-derived G-MDSCs were purified from LPS or LPS plus zVAD treated C57BL/6 mice using a Myeloid-Derived Suppressor Cell Isolation Kit. BMDMs (2×10^5 cells/well) were co-cultured with purified G-MDSCs for 12 hours. Then, BMDMs were stimulated with 100 ng/ml LPS and cultured for 24 hours. In the end, the expression levels of CD86 in the BMDMs were analyzed by flow cytometry**.**

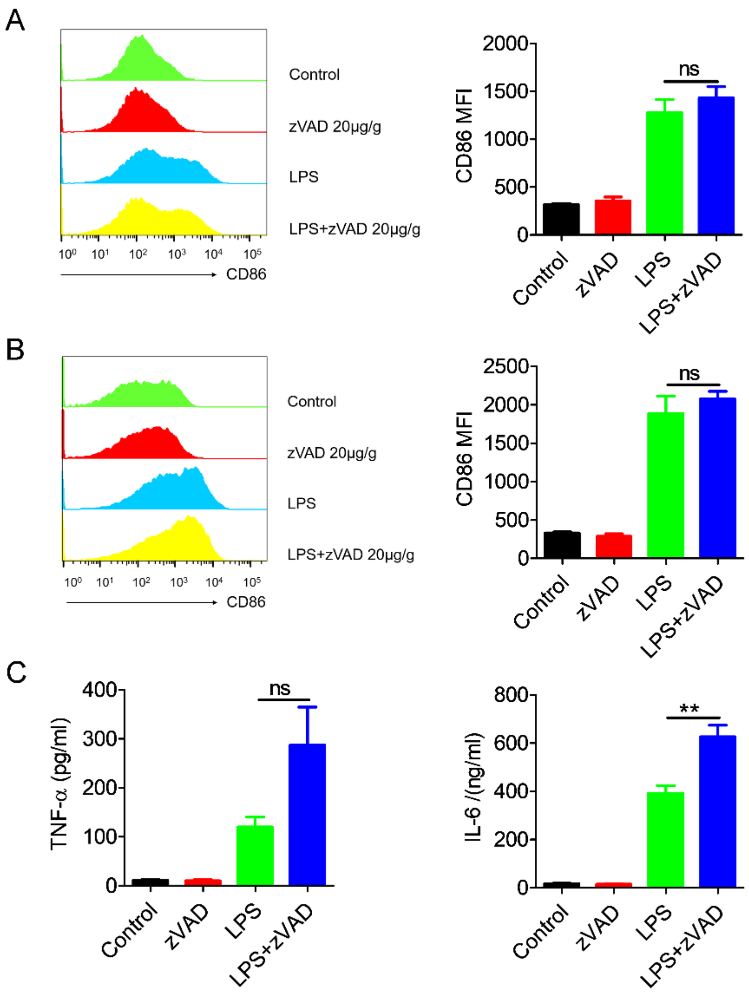

Figure S9 Post-treatment of zVAD seemly had no effect on LPS-induced endotoxin shock.

(A-C) The mice were challenged with LPS (10μg/g body weight) for 1 hour followed by zVAD (20 μg/g body weight) treatment. (A-B) After 12 hours, the expression levels of CD86 on F4/80^{+} cells (A) and CD11c^{+} cells (B) in spleens were measured for flow cytometry. (C) After 6 hours, levels of TNF-α and IL-6 in serum were measured by ELISA. Data are presented as means ± S.E.M. of triplicate measurements and are representative of three independent experiments. Error bars represent S.E.M.; **$p < 0.01$ as determined by ANOVA test, ns denotes $p > 0.05$.

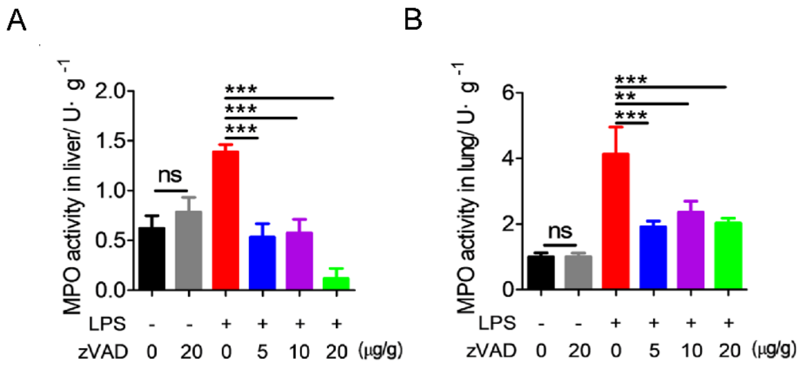

Figure S10 zVAD can decrease the infiltration of neutrophil in LPS-challenged liver and lung of mice.

C57BL/6 mice were pretreated with different doses of zVAD (5, 10, or 20 μg/g body weight) or vehicle (saline) prior to LPS challenge (10 μg/g body weight). After 12 h, liver and lung tissues were collected. The neutrophil infiltration in liver (A) and lung (B) was measured by MPO activity detection kit. The data are presented as the mean ± SEM of triplicates. Error bars represent S.E.M. ***p < 0.001, as determined by ANOVA test; ns denotes p > 0.05.
